# Long-term safety and efficacy of fostamatinib in Japanese patients with primary immune thrombocytopenia

**DOI:** 10.1007/s12185-025-03924-2

**Published:** 2025-01-28

**Authors:** Masataka Kuwana, Yoshiaki Tomiyama

**Affiliations:** 1 Department of Allergy and Rheumatology, Nippon Medical School Graduate School of Medicine, 1-1-5 Sendagi, Bunkyo-ku, Tokyo, Japan; 2 Department of Blood Transfusion, Osaka University Hospital, Osaka, Japan

**Keywords:** Fostamatinib, Phase 3 clinical trial, Primary immune thrombocytopenia, Spleen tyrosine kinase

## Abstract

Fostamatinib had superior efficacy to a placebo and acceptable safety profiles for at least 1 year in a phase 3 study of Japanese patients with primary immune thrombocytopenia. Here, we report the 3-year safety and efficacy of fostamatinib in that study. Data from 33 patients who received at least one dose of fostamatinib were analyzed. A platelet response > 50,000/µL (at two consecutive visits at least 28 days apart while receiving fostamatinib) was achieved in 16 patients (48%). The median total duration of a platelet response > 50,000/µL was 589 (range: 106–1003) days. Gastrointestinal disorders, such as diarrhea, hypertension, and hepatic enzyme elevation, were the most common fostamatinib-related adverse events. Most events occurred within 12 weeks of treatment. No thromboembolisms, treatment-related infections, or moderate or severe treatment-related bleeding events were observed. In summary, this extension study of a clinical trial found a sustained platelet response without new safety signals during 3-year treatment with fostamatinib.

**Supplementary Information:**

The online version contains supplementary material available at 10.1007/s12185-025-03924-2.

## Introduction and methods

Fostamatinib, an oral spleen tyrosine kinase (Syk) inhibitor, is an approved treatment for primary immune thrombocytopenia (ITP) [1]. Its effects are mediated via directly interference with the pathogenic autoimmune loop [2] and the suppression of autoantibody production [3].

We previously reported the results of a phase 3 clinical trial of Japanese patients with ITP (R788-1301, NCT04132050), consisting of a 24-week placebo-controlled period (period I) and a 28-week open-label period (period II), followed by an optional washout challenge for up to 4 weeks [4, 5]. Patients who completed period II were eligible to enroll in an extension study, receiving 100 mg fostamatinib, once daily, to 150 mg twice daily, until the launch of commercial fostamatinib products in Japan (period III). Here, we report the 3-year safety and efficacy profiles of fostamatinib in that study.

## Results

**Fig. 1:**
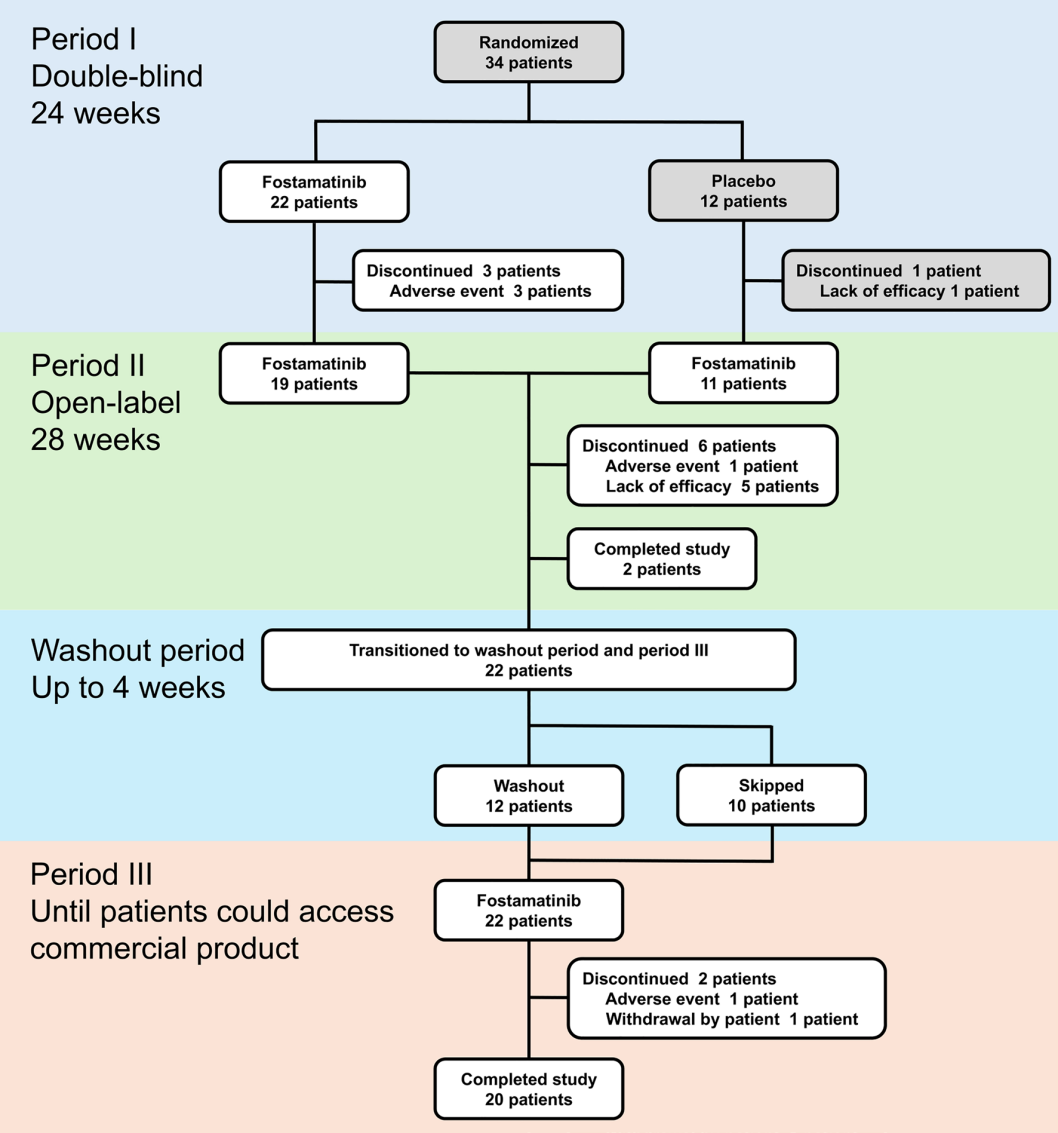
Patient flow chart. A total of 34 patients were randomized to either the fostamatinib or the placebo group. Among them, 33 patients were treated with fostamatinib at least once. One patient who received the placebo withdrew from the study during period I. Twelve patients transitioned to a washout period. Twenty-two patients transitioned to period III. Two patients exited the study after period II, and 20 completed period III

Thirty-three patients received at least one dose of fostamatinib during the study (Fig. 1); 24 completed period II, and 20 completed period III. Overall, 11/33 (33%) patients discontinued the study (adverse events [AEs] and lack of efficacy in 5 cases each, and withdrawal of consent in 1 case), which was lower than the global phase 3 trial (97/146 [66%]) [6]. There was no difference in baseline characteristics between patients who continued and discontinued fostamatinib throughout study period (Table S1).

The median (range) age of the 33 patients was 62 (25–81) years; 79% were female. The baseline platelet count was 19,000/µL (1,000–28,000/µL), and the ITP duration was 14 (1–41) years (Table S1). The duration of fostamatinib exposure was 771 (42–1184) days, and the median average daily dose was 238 (145–297) mg/day.

**Fig. 2:**
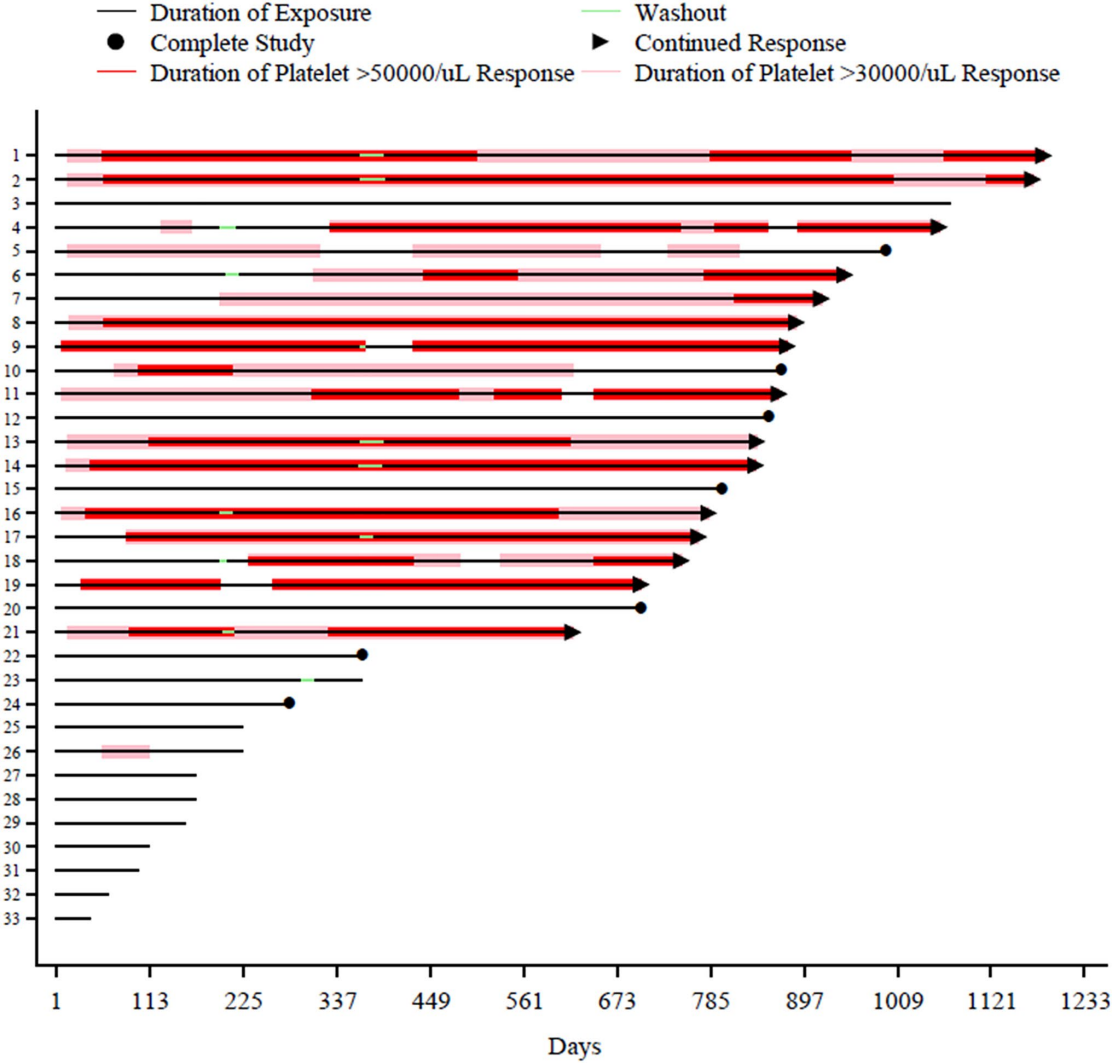
Swimmer plot with platelet response. Duration of platelet response > 50,000/µL: the maintenance start date was defined as the day after the start of study-drug administration, when the platelet count was 50,000/µL or higher for at least 28 consecutive days without the use of rescue medication. The maintenance end date was defined as the day when rescue medication was used or the day when the platelet count dropped below 50,000/µL for 28 or more consecutive days. The duration of the > 30,000/µL platelet response was defined in the same way. Data obtained after the first dose of fostamatinib are shown

A platelet response > 50,000/µL (at two consecutive visits at least 28 days apart while receiving fostamatinib), which was predefined in this trial protocol [4], was achieved in 16/33 patients (48%) (Fig. 2). The median total duration of a platelet response > 50,000/µL was 589 (106–1,003) days. A platelet response > 30,000/µL was achieved in 18 patients (55%); the median total duration of such a response was 727 (57–1,170) days. No platelet overshoots (> 400,000/µL) were observed.

Platelet counts > 50,000/µL were maintained at the end of the study in 13/16 patients. In two of the remaining three patients (#13 and #16), platelet counts were maintained > 30,000/µL without bleeding symptoms. The other patient (#10) experienced a drop in platelet counts to 10,000–20,000/µL during herpes zoster, but no bleeding was observed and the platelet count recovered spontaneously.

**Fig. 3:**
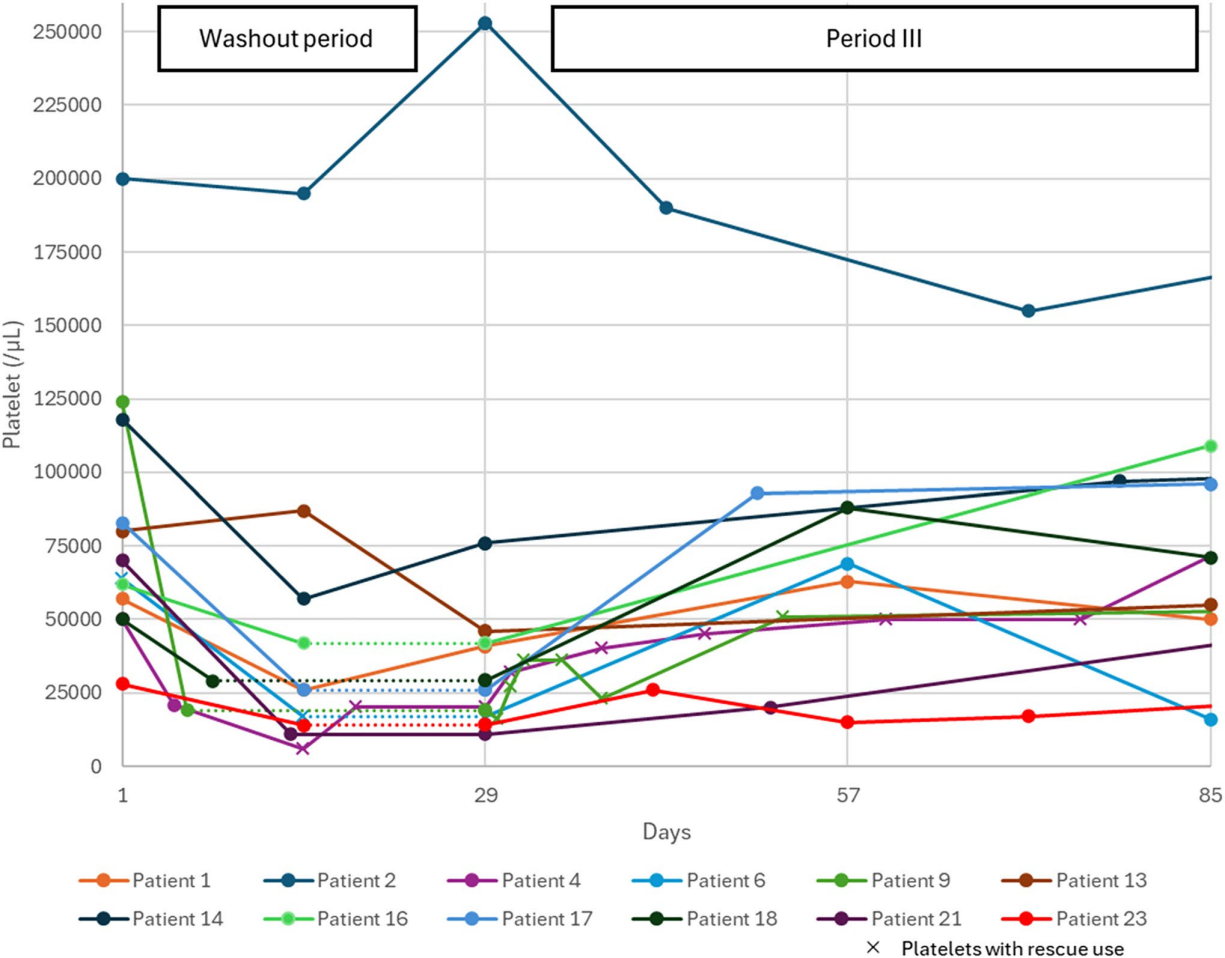
Platelet counts of patients who proceeded to the washout period and participated up to week 8 of period III. The dotted line indicates the transition to period III after the discontinuation of the washout period. Patient 2 withdrew from fostamatinib treatment at 14 days after the start of period III because of an elevated platelet count during the washout period

All 12 patients in the washout challenge had a decrease in platelet counts with the suspension of fostamatinib, but none experienced bleeding events (Fig. 3). Their platelet counts recovered during period III.

Patients were allowed to take concomitant ITP medications (glucocorticoids, azathioprine, or danazol) at a fixed dose during period I and a flexible dose after period I. Among 14 patients receiving glucocorticoid treatment at the beginning of period II, 3 underwent dosage reduction (prednisolone; 10–7.5 mg/day for 2 and 10–2 mg/day for 1), and 4 discontinued prednisolone (5, 5, 2.5, and 2 mg/day), owing to a treatment response to fostamatinib (Figure S1). Patients with a platelet count < 50,000/μL were allowed to use rescue medication. Rescue medication/treatment was administered to 13/33 (39%) patients, including glucocorticoids to 7, platelet transfusions to 5, and intravenous immunoglobulin to 4 patients. The major reasons for rescue medication/treatment were a lack of efficacy (six non-responders) and temporary use after the washout challenge (two patients).

All 33 patients experienced at least one AE (Table 1). The most common AEs (> 10% of cases) were diarrhea, hypertension, coronavirus disease 2019 (COVID-19), constipation, eczema, nasopharyngitis, and hepatic-enzyme elevation (Table S2). The majority of events were mildly (48%) or moderately (42%) severe. Treatment-related AEs that occurred in ≥ 10% of patients were diarrhea and hypertension (30% each). Most treatment-related AEs occurred before week 12 (Table S3).

**Table 1:** Adverse Events by Onset Time

	Overall			Week ≤ 12		
	n	%	Event	n	%	Event
N	33	–	–	33	–	–
Adverse Events	33	100	237	25	76	80
Treatment-related Adverse Events	24	73	74	19	58	44
Adverse Events of Interest						
Bleeding Events	7	21	15	4	12	6
Gastrointestinal Complaints	16	48	35	14	42	21
Nausea	1	3	2	1	3	1
Vomiting	2	6	2	0	0	0
Non-Infectious Diarrhea	13	39	26	11	33	18
Abdominal Pain	5	15	5	2	6	2
Infection	19	58	34	2	6	2
Hypertension	13	39	17	10	30	12
Neutropenia	6	18	11	3	9	3
Drug-Related Hepatic Disorders	11	33	20	8	24	11
Thrombosis, embolism, and thromboembolism	0	0	0	0	0	0

No deaths occurred during the study. Serious AEs were observed in eight patients (24%), including COVID-19, cellulitis, gastroenteritis, herpes zoster, pericoronitis, thrombocytopenia, atrial fibrillation, diarrhea, large-intestinal polyps, autoimmune hepatitis, lumbar-spinal stenosis, and radial fracture (one each). Serious treatment-related AEs were observed in two patients (6%): thrombocytopenia and diarrhea (one each). AEs leading to drug withdrawal occurred in five patients (15%). Hepatic enzyme elevation (two patients) was the only AE leading to study-drug withdrawal of more than one patient.

The most frequent AEs were gastrointestinal disorders, hypertension, and hepatic enzyme elevation. Most of these events occurred within 12 weeks of treatment (Table 1 and Table S3). Only one of seven bleeding events, a mild case, was considered treatment-related. Thromboses, embolisms, and thromboembolisms were not observed during the study period. Infection was observed in 19 patients (58%), none of which were deemed related to fostamatinib. The infection was mild in 12 patients and moderate in 7 patients. None of the infections led to withdrawal from the study. COVID-19 occurred in 6 patients (18%). One patient was hospitalized for COVID-19 per their request, although the event was moderately severe. In that patient, COVID-19 occurred 592 days after the start of fostamatinib treatment; they were treated with remdesivir and recovered two days after admission. The investigator judged the disease unrelated with fostamatinib treatment because the patient had apparently had contact with a person with COVID-19 prior to the onset of the event.

## Discussion

The goal for ITP treatment is maintenance of platelet counts > 30,000/μL to avoid serious bleeding events [7, 8]. In this 3-year extension of a clinical trial of Japanese patients treated with fostamatinib, 16/33 had a platelet response > 50,000/μL and 18/33 > 30,000/μL throughout. Some responders received rescue treatment after the washout challenge, but platelet counts recovered after restart of fostamatinib, and none of them required rescue treatment during treatment with fostamatinib. The sustained efficacy in patients who initially respond to fostamatinib was previously observed in a global phase 3 study for 5 years [9]. In this regard, TPO-RAs may cause substantial fluctuations in platelet counts that are difficult to manage [10, 11], and the 1- and 5-year efficacy rates of maintaining platelet count > 50,000/μL were 38% and 21%, respectively, in the rituximab trial [12].

Gastrointestinal disorders, such as diarrhea, hypertension, and hepatic enzyme elevation, are the most common fostamatinib-related AEs [5, 9]. In this study, most events occurred within 12 weeks, and no new safety risks were observed over 3 years. Thus, once these side effects are controlled, fostamatinib is safe and well-tolerated in long-term use.

Although fostamatinib potentially suppresses the innate and acquired immune response [13], in this study, no severe infection or infection that led to discontinuation of fostamatinib were observed throughout the 3 years. No infection was considered treatment related by the investigators. Half of the patients who used glucocorticoids were able to reduce or discontinue the glucocorticoid dose after responding to fostamatinib, reducing the risk of infection. In total, fostamatinib is unlikely to increase the infection risk or severity.

Patients with ITP have a 3- to 4-rfold higher risk of thromboembolism than healthy individuals [14], which is further increased by treatment with TPO-RAs or splenectomy [15]. Syk inhibition may inhibit thrombus formation [16, 17]. In a 5-year, global phase 3 study of fostamatinib, only one thromboembolic event was observed (0.7%, 0.44/100 patient-years) [9]. In this 3-year study, no such events were observed. However, further investigations, such as post-marketing surveillance of patients, including those with a thrombotic risk, are warranted.

This study had several limitations. First, only 33 patients were analyzed. Second, the maximum treatment period was 3 years. A longer study with a larger sample is needed to verify our results. In conclusion, this study of fostamatinib treatment for ITP revealed a platelet response for up to 3 years without new safety signals.

## Supplementary Information

Below is the link to the electronic supplementary material.

Supplementary file1 (DOCX 3688 KB)

## Data Availability

The data that support the finding of this study are not available owing to a lack of patient consent to release them to the public.
