# Supplementary material for: Long-term safety and efficacy of fostamatinib in Japanese patients with primary immune thrombocytopenia
Source: Int J Hematol. 2025 Jan 28;121(3):356–62. doi: 10.1007/s12185-025-03924-2 (PMC11861395; doi:10.1007/s12185-025-03924-2)

Supplementary Appendix

Article: Long-term safety and efficacy of fostamatinib in Japanese patients with primary immune thrombocytopenia

Table S1 Baseline characteristics of patients

| Characteristics | Overall^c^  N = 33 | Completed^d^  N=22 | Discontinued^e^  N=11 |
| --- | --- | --- | --- |
| Sex, n (%) |  |  |  |
| Male | 7 (21) | 4 (18) | 3 (27) |
| Female | 26 (79) | 18 (82) | 8 (73) |
| Age, years^a^ | 62 (25, 81) | 62 (31, 76) | 67 (25, 81) |
| Baseline platelet count, × 10^3^/μL^a^ | 19 (1, 28) | 17 (1, 27) | 21 (3, 28) |
| Duration of ITP, years^a^ | 14 (1, 41) | 14 (1, 41) | 5 (1, 27) |
| Splenectomy, n (%) | 7 (21) | 6 (27) | 1 (9) |
| Lines of therapy, n (%) |  |  |  |
| 2 | 14 (42) | 9 (41) | 5 (45) |
| ≥ 3 | 19 (58) | 13 (59) | 6 (55) |
| Number of prior ITP medications^a^ | 2 (1, 10) | 2 (1, 10) | 2 (1, 6) |
| Prior ITP medications^b^, n (%) |  |  |  |
| Glucocorticoids | 32 (97) | 22 (100) | 10 (91) |
| Rituximab | 4 (12) | 3 (14) | 1 (9) |
| Thrombopoietin-receptor agonists | 17 (52) | 12 (55) | 5 (45) |
| Intravenous immunoglobulin | 12 (36) | 7 (32) | 5 (45) |
| Immunosuppressants | 4 (12) | 3 (14) | 1 (9) |
| Others | 5 (15) | 4 (18) | 1 (9) |

ITP, immune thrombocytopenia; n, number of patients

^a^Median (range).

^b^Some patients used more than two ITP medications.

^c^Patients treated with fostamatinib at least once.

^d^ Patients who completed the study.

^e^ Patients who discontinued the study.

Table S2 Adverse Events

|  | Adverse Events | | | Treatment-related  Adverse Events | | |
| --- | --- | --- | --- | --- | --- | --- |
|  | n | % | e | n | % | e |
| N | 33 | --- | --- | 33 | --- | --- |
| Total | 33 | 100 | 237 | 24 | 73 | 74 |
| Infections and infestations | 19 | 58 | 34 | 0 | 0 | 0 |
| COVID-19 | 6 | 18 | 6 | 0 | 0 | 0 |
| Cellulitis | 1 | 3 | 1 | 0 | 0 | 0 |
| Coronavirus infection | 1 | 3 | 1 | 0 | 0 | 0 |
| Cystitis | 2 | 6 | 2 | 0 | 0 | 0 |
| Diverticulitis | 1 | 3 | 1 | 0 | 0 | 0 |
| Gastroenteritis | 2 | 6 | 2 | 0 | 0 | 0 |
| Gingivitis | 1 | 3 | 1 | 0 | 0 | 0 |
| Herpes simplex virus infection | 1 | 3 | 1 | 0 | 0 | 0 |
| Herpes zoster | 3 | 9 | 3 | 0 | 0 | 0 |
| Nasopharyngitis | 4 | 12 | 5 | 0 | 0 | 0 |
| Paronychia | 2 | 6 | 3 | 0 | 0 | 0 |
| Pericoronitis | 1 | 3 | 1 | 0 | 0 | 0 |
| Periodontitis | 1 | 3 | 1 | 0 | 0 | 0 |
| Pulpitis | 1 | 3 | 1 | 0 | 0 | 0 |
| Sinusitis | 1 | 3 | 1 | 0 | 0 | 0 |
| Tinea infection | 1 | 3 | 1 | 0 | 0 | 0 |
| Urinary-tract infection | 1 | 3 | 1 | 0 | 0 | 0 |
| Viral infection | 2 | 6 | 2 | 0 | 0 | 0 |
| Neoplasms: benign, malignant and unspecified (incl. cysts and polyps) | 2 | 6 | 2 | 1 | 3 | 1 |
| Lipoma | 1 | 3 | 1 | 0 | 0 | 0 |
| Myelodysplastic syndrome | 1 | 3 | 1 | 1 | 3 | 1 |
| Blood and lymphatic-system disorders | 4 | 12 | 9 | 3 | 9 | 5 |
| Leukopenia | 1 | 3 | 1 | 1 | 3 | 1 |
| Neutropenia | 3 | 9 | 7 | 2 | 6 | 3 |
| Thrombocytopenia | 1 | 3 | 1 | 1 | 3 | 1 |
| Endocrine disorders | 1 | 3 | 1 | 0 | 0 | 0 |
| Hypothyroidism | 1 | 3 | 1 | 0 | 0 | 0 |
| Metabolism and nutrition disorders | 5 | 15 | 7 | 1 | 3 | 2 |
| Decreased appetite | 1 | 3 | 1 | 0 | 0 | 0 |
| Dehydration | 1 | 3 | 1 | 0 | 0 | 0 |
| Hyperglycemia | 1 | 3 | 1 | 0 | 0 | 0 |
| Hypoalbuminemia | 2 | 6 | 2 | 1 | 3 | 1 |
| Hypoglycemia | 1 | 3 | 1 | 0 | 0 | 0 |
| Hypoproteinemia | 1 | 3 | 1 | 1 | 3 | 1 |
| Psychiatric disorders | 2 | 6 | 2 | 0 | 0 | 0 |
| Affect lability | 1 | 3 | 1 | 0 | 0 | 0 |
| Insomnia | 1 | 3 | 1 | 0 | 0 | 0 |
| Nervous-system disorders | 3 | 9 | 3 | 0 | 0 | 0 |
| Cervicobrachial syndrome | 1 | 3 | 1 | 0 | 0 | 0 |
| Dizziness | 1 | 3 | 1 | 0 | 0 | 0 |
| Trigeminal neuralgia | 1 | 3 | 1 | 0 | 0 | 0 |
| Eye disorders | 6 | 18 | 9 | 0 | 0 | 0 |
| Asthenopia | 1 | 3 | 1 | 0 | 0 | 0 |
| Blepharitis | 1 | 3 | 1 | 0 | 0 | 0 |
| Conjunctival hemorrhage | 1 | 3 | 1 | 0 | 0 | 0 |
| Conjunctival hyperemia | 1 | 3 | 1 | 0 | 0 | 0 |
| Conjunctivitis, allergic | 1 | 3 | 1 | 0 | 0 | 0 |
| Macular fibrosis | 1 | 3 | 1 | 0 | 0 | 0 |
| Ocular discomfort | 1 | 3 | 1 | 0 | 0 | 0 |
| Punctate keratitis | 1 | 3 | 1 | 0 | 0 | 0 |
| Vitreous floaters | 1 | 3 | 1 | 0 | 0 | 0 |
| Ear and labyrinth disorders | 3 | 9 | 4 | 0 | 0 | 0 |
| Auricular swelling | 1 | 3 | 1 | 0 | 0 | 0 |
| Eustachian tube, patulous | 1 | 3 | 1 | 0 | 0 | 0 |
| Meniere’s disease | 1 | 3 | 1 | 0 | 0 | 0 |
| Sudden hearing loss | 1 | 3 | 1 | 0 | 0 | 0 |
| Cardiac disorders | 1 | 3 | 1 | 0 | 0 | 0 |
| Atrial fibrillation | 1 | 3 | 1 | 0 | 0 | 0 |
| Vascular disorders | 12 | 36 | 15 | 10 | 30 | 13 |
| Hypertension | 11 | 33 | 14 | 10 | 30 | 13 |
| Orthostatic hypotension | 1 | 3 | 1 | 0 | 0 | 0 |
| Respiratory, thoracic, and mediastinal disorders | 3 | 9 | 5 | 0 | 0 | 0 |
| Cough | 1 | 3 | 1 | 0 | 0 | 0 |
| Epistaxis | 2 | 6 | 3 | 0 | 0 | 0 |
| Oropharyngeal pain | 1 | 3 | 1 | 0 | 0 | 0 |
| Gastrointestinal disorders | 21 | 64 | 48 | 11 | 33 | 24 |
| Abdominal discomfort | 3 | 9 | 3 | 1 | 3 | 1 |
| Abdominal pain | 1 | 3 | 1 | 0 | 0 | 0 |
| Abdominal pain, upper | 1 | 3 | 1 | 0 | 0 | 0 |
| Constipation | 5 | 15 | 5 | 0 | 0 | 0 |
| Dental caries | 1 | 3 | 1 | 0 | 0 | 0 |
| Diarrhea | 13 | 39 | 26 | 10 | 30 | 20 |
| Feces, soft | 1 | 3 | 1 | 0 | 0 | 0 |
| Large intestinal polyp | 1 | 3 | 1 | 0 | 0 | 0 |
| Mouth hemorrhage | 1 | 3 | 1 | 1 | 3 | 1 |
| Nausea | 1 | 3 | 2 | 1 | 3 | 1 |
| Stomatitis | 3 | 9 | 3 | 1 | 3 | 1 |
| Toothache | 1 | 3 | 1 | 0 | 0 | 0 |
| Vomiting | 2 | 6 | 2 | 0 | 0 | 0 |
| Hepatobiliary disorders | 4 | 12 | 6 | 2 | 6 | 3 |
| Autoimmune hepatitis | 1 | 3 | 1 | 0 | 0 | 0 |
| Hepatic cyst | 1 | 3 | 1 | 0 | 0 | 0 |
| Hepatic function, abnormal | 2 | 6 | 3 | 2 | 6 | 3 |
| Hepatic steatosis | 1 | 3 | 1 | 0 | 0 | 0 |
| Skin and subcutaneous-tissue disorders | 15 | 45 | 22 | 1 | 3 | 1 |
| Dry skin | 1 | 3 | 1 | 0 | 0 | 0 |
| Eczema | 5 | 15 | 5 | 0 | 0 | 0 |
| Eczema, asteatotic | 1 | 3 | 1 | 0 | 0 | 0 |
| Erythema | 2 | 6 | 2 | 0 | 0 | 0 |
| Hemorrhage, subcutaneous | 3 | 9 | 4 | 0 | 0 | 0 |
| Pruritus | 2 | 6 | 3 | 0 | 0 | 0 |
| Purpura | 1 | 3 | 1 | 1 | 3 | 1 |
| Rash | 2 | 6 | 2 | 0 | 0 | 0 |
| Rash, erythematous | 1 | 3 | 1 | 0 | 0 | 0 |
| Rosacea | 1 | 3 | 1 | 0 | 0 | 0 |
| Seborrheic dermatitis | 1 | 3 | 1 | 0 | 0 | 0 |
| Musculoskeletal and connective-tissue disorders | 7 | 21 | 14 | 0 | 0 | 0 |
| Arthralgia | 2 | 6 | 4 | 0 | 0 | 0 |
| Back pain | 2 | 6 | 2 | 0 | 0 | 0 |
| Joint contracture | 1 | 3 | 1 | 0 | 0 | 0 |
| Lumbar-spinal stenosis | 1 | 3 | 1 | 0 | 0 | 0 |
| Muscle spasms | 1 | 3 | 1 | 0 | 0 | 0 |
| Muscular weakness | 1 | 3 | 1 | 0 | 0 | 0 |
| Neck pain | 1 | 3 | 1 | 0 | 0 | 0 |
| Osteoarthritis | 1 | 3 | 1 | 0 | 0 | 0 |
| Pain in extremity | 2 | 6 | 2 | 0 | 0 | 0 |
| Renal and urinary disorders | 2 | 6 | 2 | 0 | 0 | 0 |
| Hematuria | 1 | 3 | 1 | 0 | 0 | 0 |
| Hypertonic bladder | 1 | 3 | 1 | 0 | 0 | 0 |
| Reproductive-system and breast disorders | 2 | 6 | 2 | 0 | 0 | 0 |
| Menopausal symptoms | 1 | 3 | 1 | 0 | 0 | 0 |
| Menorrhagia | 1 | 3 | 1 | 0 | 0 | 0 |
| General disorders and administration-site conditions | 7 | 21 | 10 | 1 | 3 | 2 |
| Face edema | 1 | 3 | 1 | 0 | 0 | 0 |
| Incarcerated hernia | 1 | 3 | 1 | 0 | 0 | 0 |
| Malaise | 1 | 3 | 1 | 0 | 0 | 0 |
| Edema | 1 | 3 | 1 | 0 | 0 | 0 |
| Edema, peripheral | 2 | 6 | 3 | 1 | 3 | 2 |
| Pain | 1 | 3 | 1 | 0 | 0 | 0 |
| Pyrexia | 1 | 3 | 1 | 0 | 0 | 0 |
| Swelling | 1 | 3 | 1 | 0 | 0 | 0 |
| Investigations | 15 | 45 | 30 | 12 | 36 | 23 |
| Alanine aminotransferase, increased | 2 | 6 | 3 | 2 | 6 | 3 |
| Aspartate aminotransferase, increased | 2 | 6 | 3 | 2 | 6 | 3 |
| Blood bilirubin, increased | 1 | 3 | 1 | 1 | 3 | 1 |
| Blood creatine phosphokinase, increased | 2 | 6 | 3 | 0 | 0 | 0 |
| Blood lactate dehydrogenase, increased | 1 | 3 | 1 | 1 | 3 | 1 |
| Blood pressure, increased | 2 | 6 | 3 | 2 | 6 | 3 |
| Hepatic enzyme, increased | 1 | 3 | 1 | 1 | 3 | 1 |
| Liver-function test result, abnormal | 1 | 3 | 1 | 1 | 3 | 1 |
| Liver-function test result, increased | 4 | 12 | 5 | 3 | 9 | 3 |
| Lymphocyte count, increased | 1 | 3 | 1 | 1 | 3 | 1 |
| Neutrophil count, decreased | 3 | 9 | 4 | 3 | 9 | 4 |
| Platelet count, decreased | 1 | 3 | 1 | 0 | 0 | 0 |
| Weight, increased | 1 | 3 | 1 | 0 | 0 | 0 |
| White-blood-cell count, decreased | 1 | 3 | 2 | 1 | 3 | 2 |
| Injury, poisoning, and procedural complications | 6 | 18 | 11 | 0 | 0 | 0 |
| Allergic transfusion reaction | 1 | 3 | 1 | 0 | 0 | 0 |
| Contusion | 1 | 3 | 2 | 0 | 0 | 0 |
| Heat exhaustion | 1 | 3 | 1 | 0 | 0 | 0 |
| Heat stroke | 1 | 3 | 1 | 0 | 0 | 0 |
| Limb injury | 1 | 3 | 1 | 0 | 0 | 0 |
| Pharyngeal injury | 1 | 3 | 1 | 0 | 0 | 0 |
| Radius fracture | 1 | 3 | 1 | 0 | 0 | 0 |
| Vaccination complication | 1 | 3 | 2 | 0 | 0 | 0 |
| Wound hemorrhage | 1 | 3 | 1 | 0 | 0 | 0 |

COVID-19 = coronavirus disease 2019, e = Number of Events

Table S3 Adverse Events by Onset time

|  | Overall | | | Week ≤12 | | | Week >12, –≤24 | | | Week >24 ≤36 | | | Week >36 ≤48 | | | Week >48 ≤72 | | | Week >72 | | |
| --- | --- | --- | --- | --- | --- | --- | --- | --- | --- | --- | --- | --- | --- | --- | --- | --- | --- | --- | --- | --- | --- |
|  | n | % | e | n | % | e | n | % | e | n | % | e | n | % | e | n | % | e | n | % | e |
| N | 33 | --- | --- | 33 | --- | --- | 31 | --- | --- | 26 | --- | --- | 24 | --- | --- | 23 | --- | --- | 21 | --- | --- |
| Adverse Events | 33 | 100 | 237 | 25 | 76 | 80 | 18 | 58 | 37 | 16 | 62 | 28 | 9 | 38 | 16 | 10 | 43 | 25 | 16 | 76 | 48 |
| Treatment-related Adverse Events | 24 | 73 | 74 | 19 | 58 | 44 | 10 | 32 | 14 | 3 | 12 | 4 | 3 | 13 | 5 | 4 | 17 | 4 | 3 | 14 | 3 |
| Adverse Events of Interest |  |  |  |  |  |  |  |  |  |  |  |  |  |  |  |  |  |  |  |  |  |
| Bleeding Events | 7 | 21 | 15 | 4 | 12 | 6 | 0 | 0 | 0 | 0 | 0 | 0 | 2 | 8 | 2 | 1 | 4 | 1 | 2 | 10 | 6 |
| Gastrointestinal Complaints | 16 | 48 | 35 | 14 | 42 | 21 | 6 | 19 | 6 | 1 | 4 | 1 | 1 | 4 | 1 | 4 | 17 | 4 | 2 | 10 | 2 |
| Nausea | 1 | 3 | 2 | 1 | 3 | 1 | 0 | 0 | 0 | 0 | 0 | 0 | 1 | 4 | 1 | 0 | 0 | 0 | 0 | 0 | 0 |
| Vomiting | 2 | 6 | 2 | 0 | 0 | 0 | 1 | 3 | 1 | 0 | 0 | 0 | 0 | 0 | 0 | 1 | 4 | 1 | 0 | 0 | 0 |
| Non-Infectious Diarrhea | 13 | 39 | 26 | 11 | 33 | 18 | 4 | 13 | 4 | 0 | 0 | 0 | 0 | 0 | 0 | 2 | 9 | 2 | 2 | 10 | 2 |
| Abdominal Pain | 5 | 15 | 5 | 2 | 6 | 2 | 1 | 3 | 1 | 1 | 4 | 1 | 0 | 0 | 0 | 1 | 4 | 1 | 0 | 0 | 0 |
| Infection | 19 | 58 | 34 | 2 | 6 | 2 | 7 | 23 | 8 | 5 | 19 | 6 | 1 | 4 | 1 | 3 | 13 | 4 | 9 | 43 | 13 |
| Hypertension | 13 | 39 | 17 | 10 | 30 | 12 | 1 | 3 | 1 | 2 | 8 | 2 | 1 | 4 | 1 | 1 | 4 | 1 | 0 | 0 | 0 |
| Neutropenia | 6 | 18 | 11 | 3 | 9 | 3 | 2 | 6 | 2 | 0 | 0 | 0 | 1 | 4 | 1 | 2 | 9 | 2 | 2 | 10 | 3 |
| Drug-Related Hepatic Disorders | 11 | 33 | 20 | 8 | 24 | 11 | 3 | 10 | 4 | 2 | 8 | 4 | 0 | 0 | 0 | 1 | 4 | 1 | 0 | 0 | 0 |
| Thrombosis, embolism, and thromboembolism | 0 | 0 | 0 | 0 | 0 | 0 | 0 | 0 | 0 | 0 | 0 | 0 | 0 | 0 | 0 | 0 | 0 | 0 | 0 | 0 | 0 |

e = Number of Events

Figure S1 Platelets and ITP Treatment by Patient

Patient 1


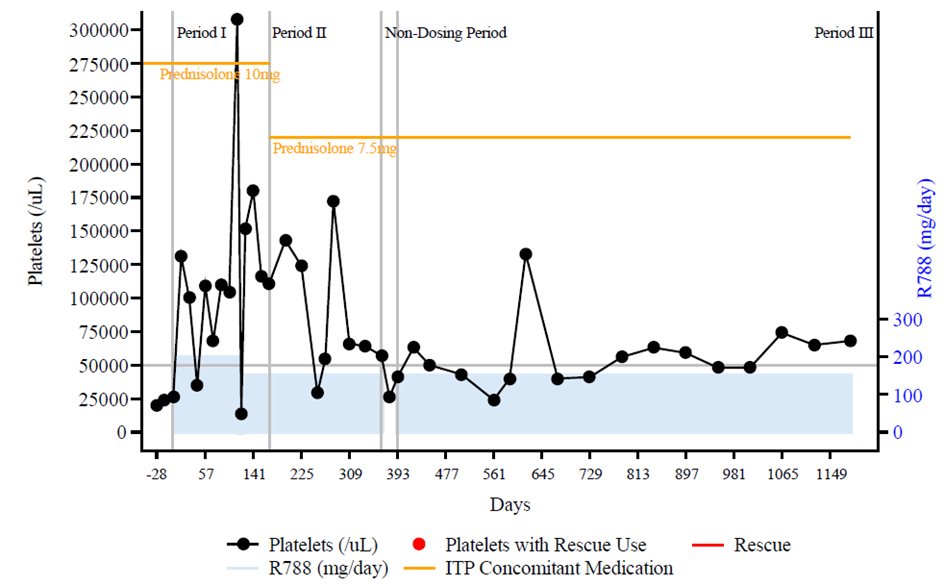


This patient used 10 mg prednisolone from day −56 to 169 and 7.5 mg from day 170.

Patient 2


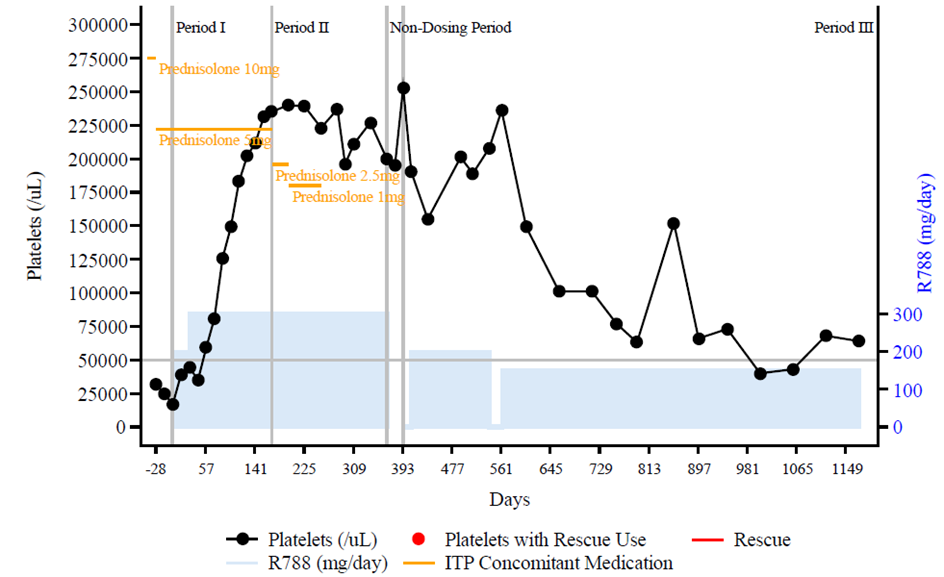


This patient used 10 mg prednisolone from day −42 to −28, 5 mg from day −27 to 170, 2.5 mg from day 171 to 198, and 1 mg from day 199 to 254. Thereafter, this patient discontinued prednisolone.

Patient 3


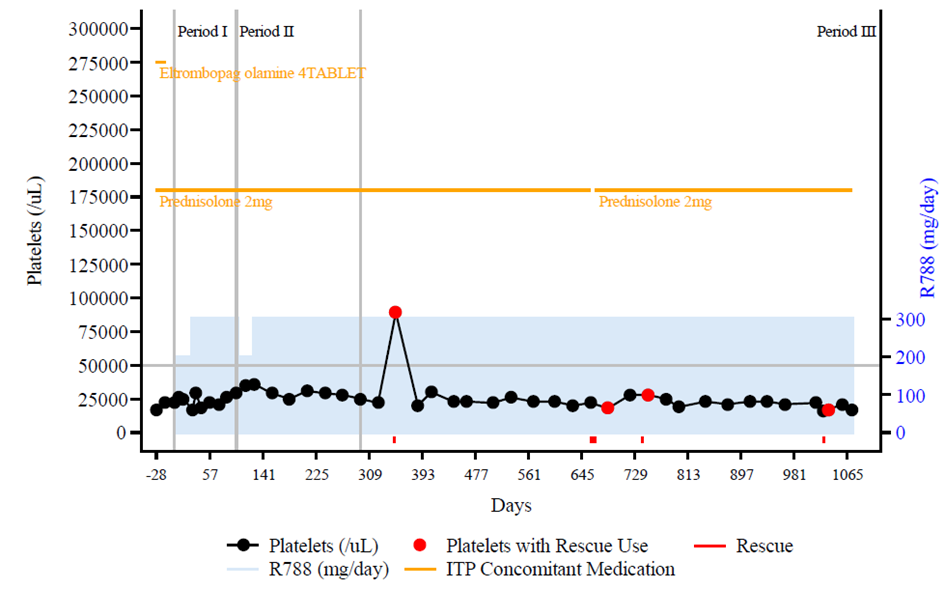


This patient used 2 mg prednisolone from day −28 to 659 and from day 667 to 1072.

Patient 4


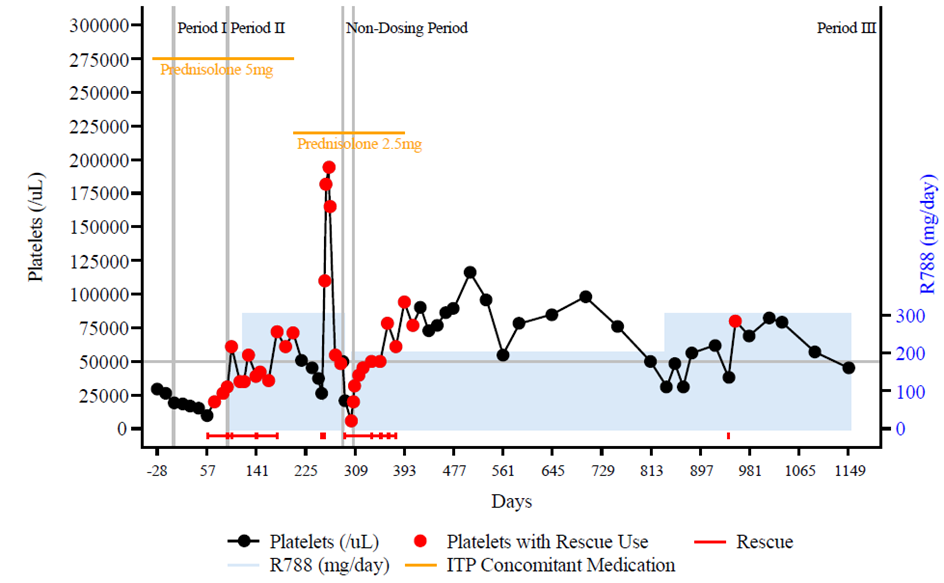


This patient used 5 mg prednisolone from day −35 to 204 and 2.5 mg from day 205 to 393. Thereafter, this patient discontinued prednisolone.

Patient 5


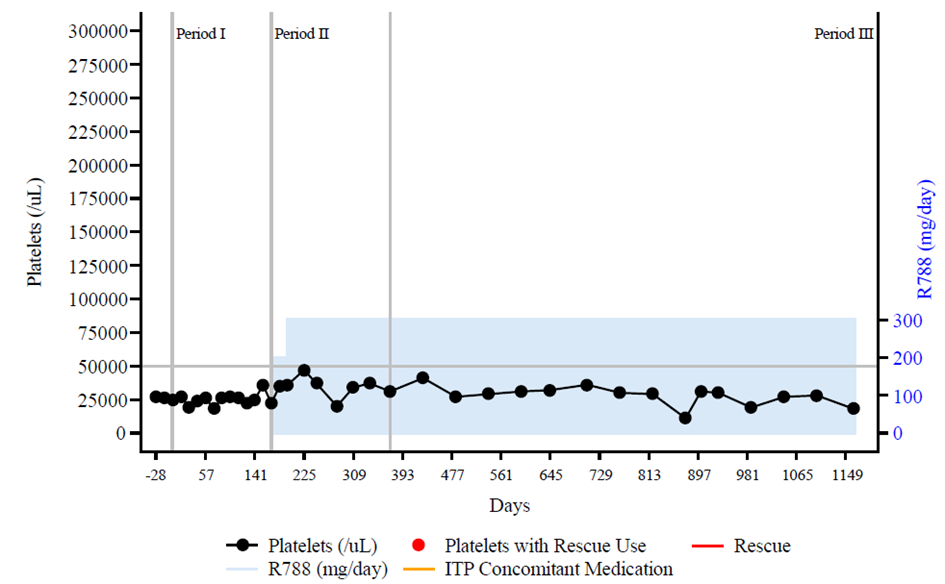


Patient 6


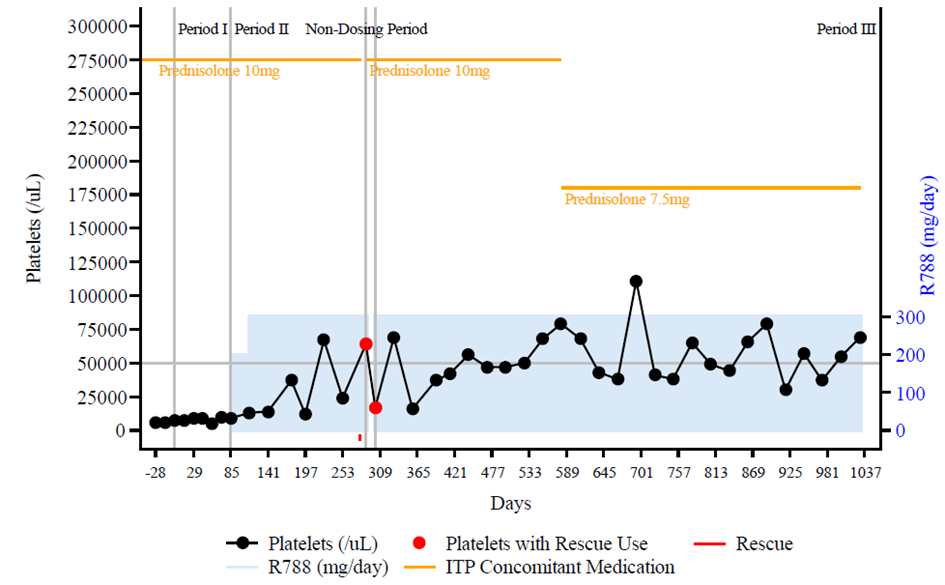


This patient used 10 mg prednisolone from day −49 to 280 and day 289 to 393, and 7.5 mg from day 582.

Patient 7


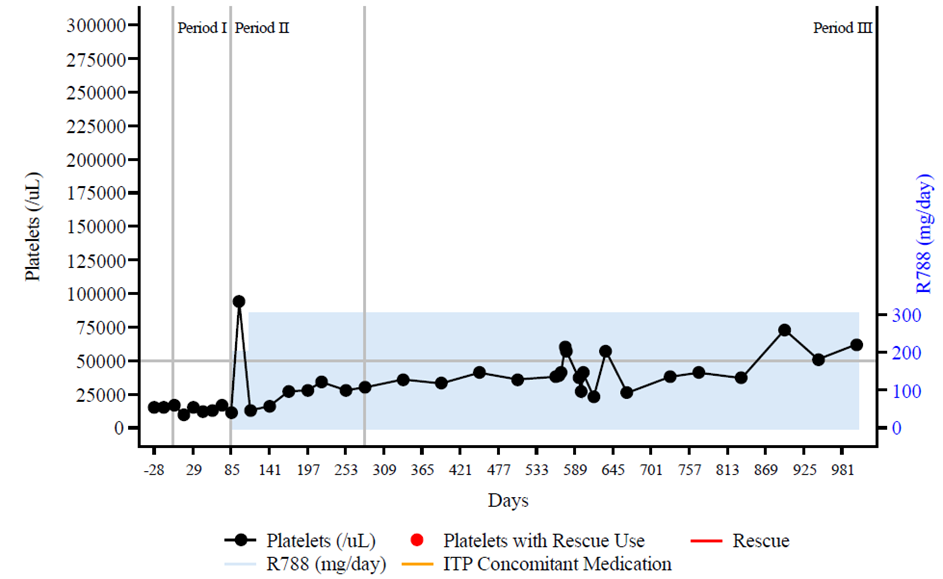


Patient 8


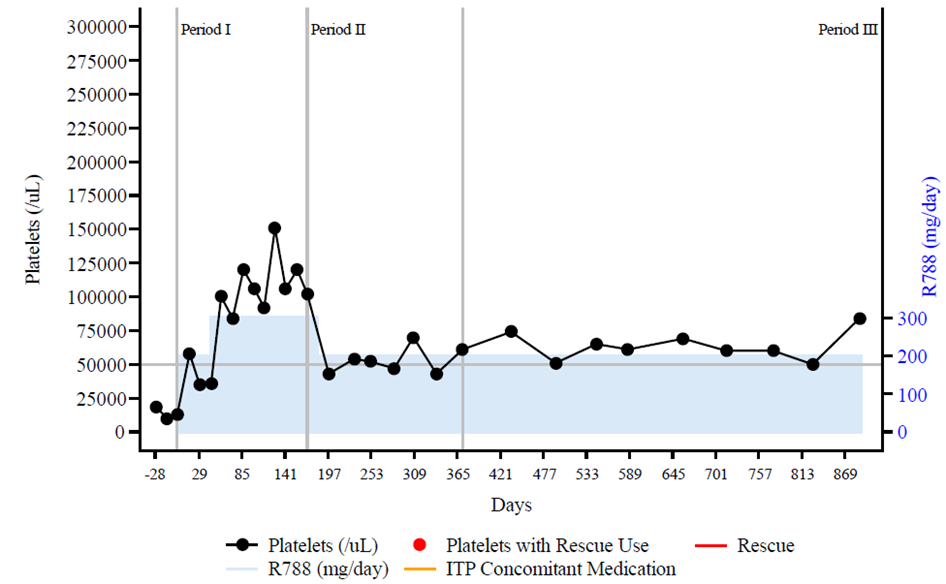


Patient 9


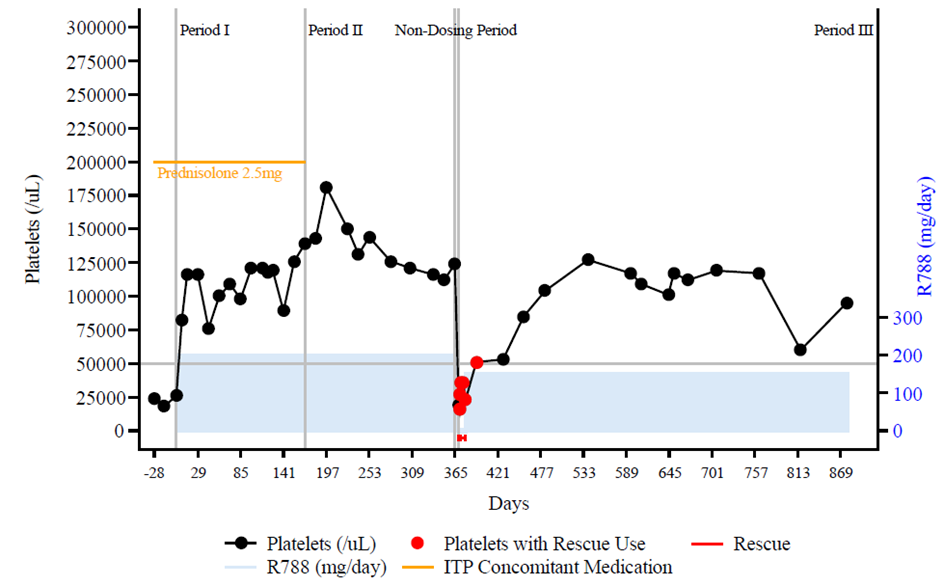


This patient used 2.5 mg prednisolone from day −28 to 169. Thereafter, this patient discontinued prednisolone.

Patient 10


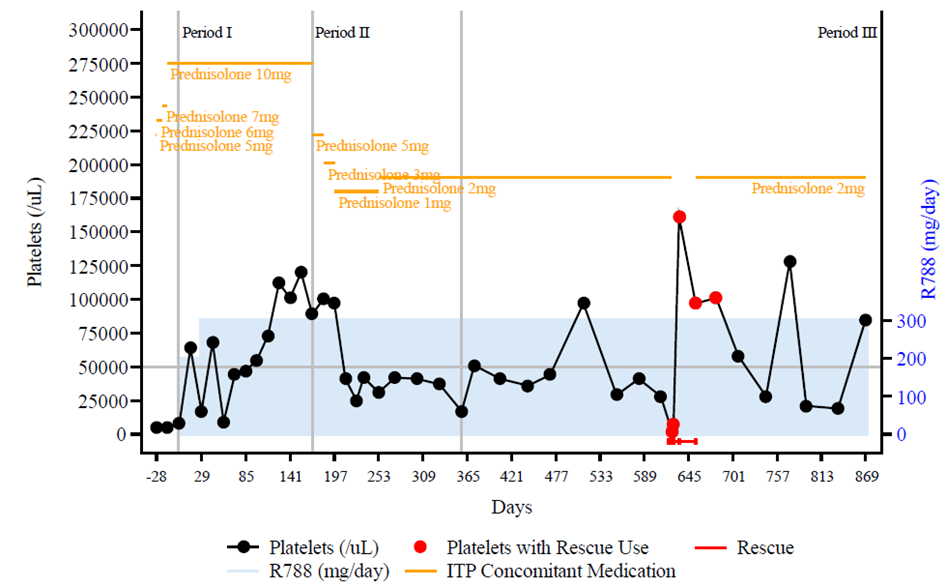


This patient used 6 mg prednisolone from day −27 to −21, 7 mg from day −20 to −15, 10 mg from day −14 to 169, 5 mg from day 170 to 183, 3 mg from day 184 to 197, 1 mg from day 198 to 253, and 2 mg from day 254 to 623 and day 655 to 869.

Patient 11


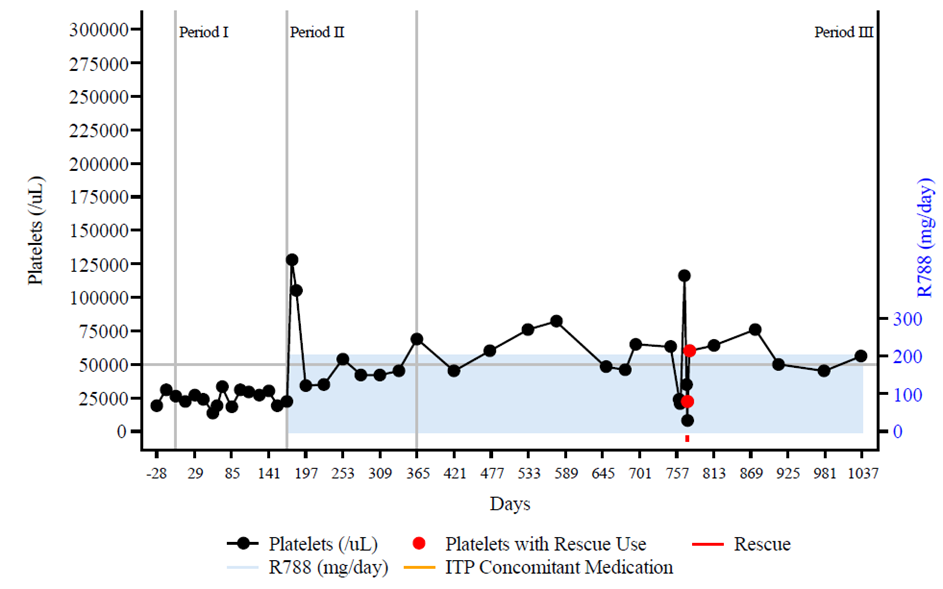


Patient 12


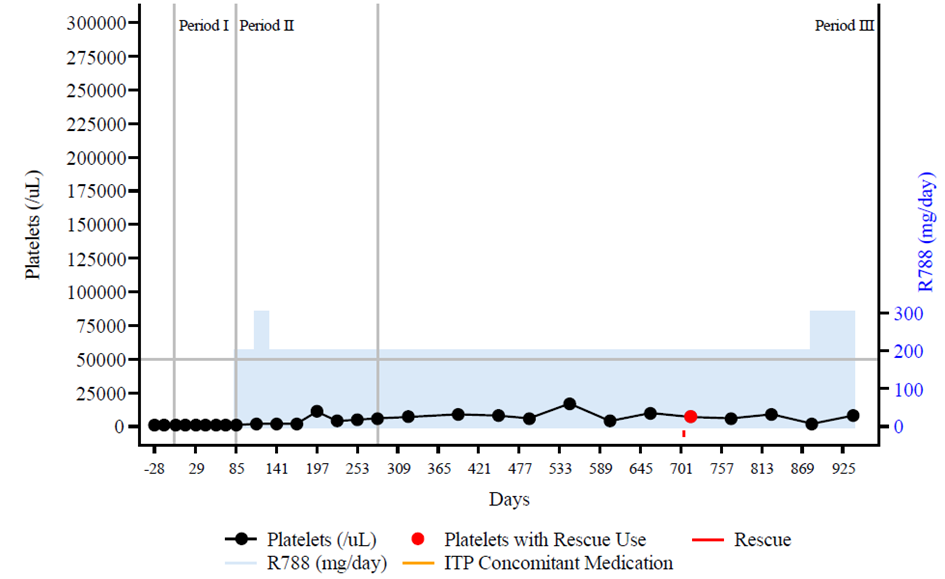


Patient 13


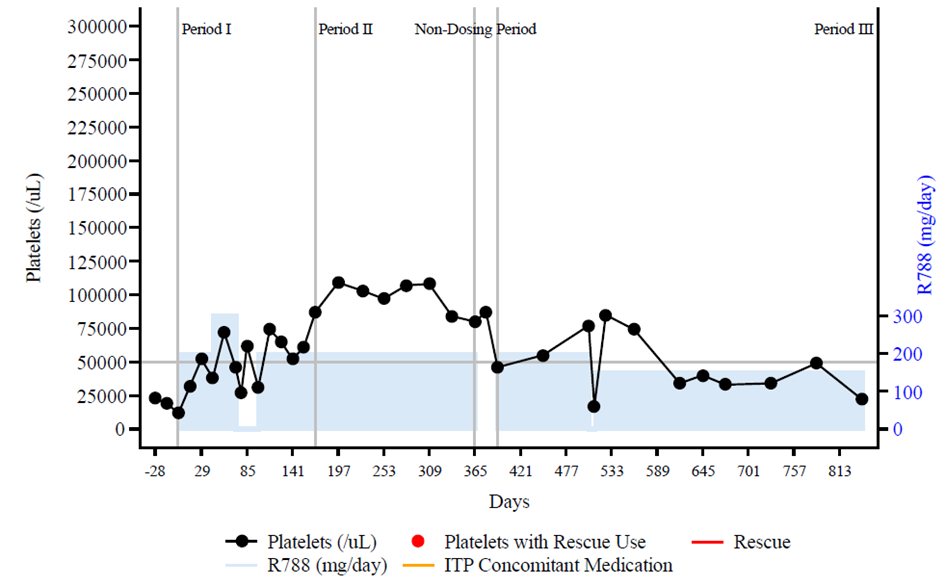


Patient 14


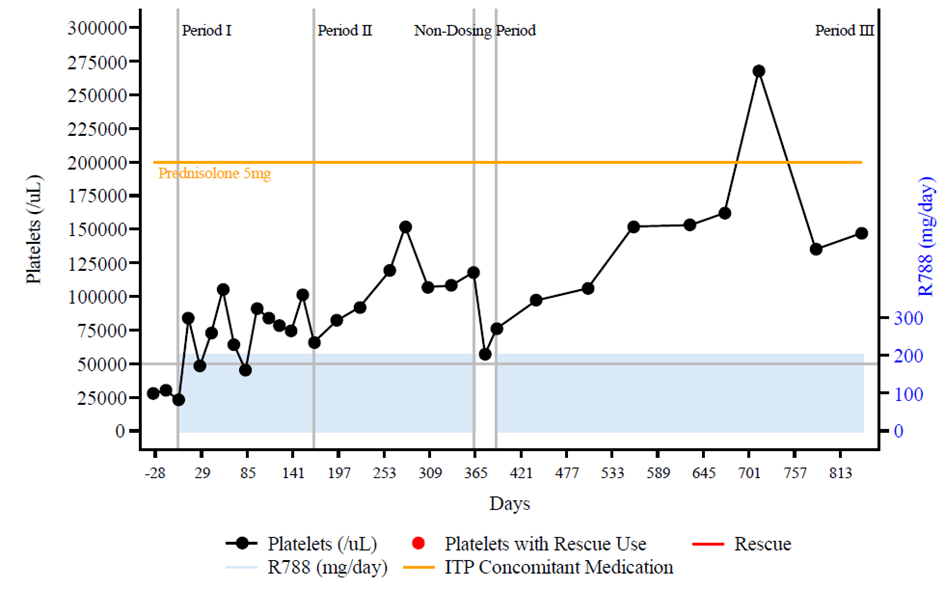


This patient used 5 mg prednisolone throughout the study.

Patient 15


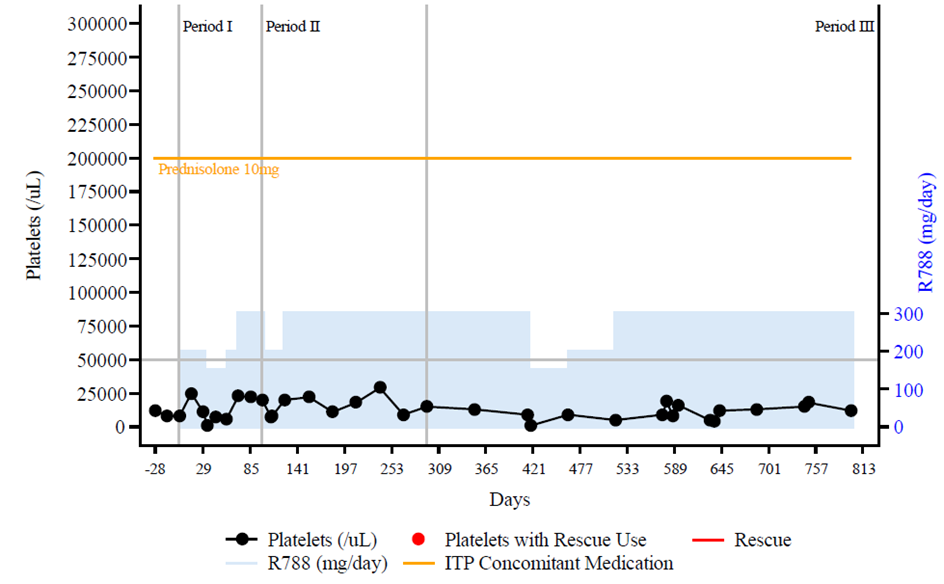


This patient used 10 mg prednisolone throughout the study.

Patient 16


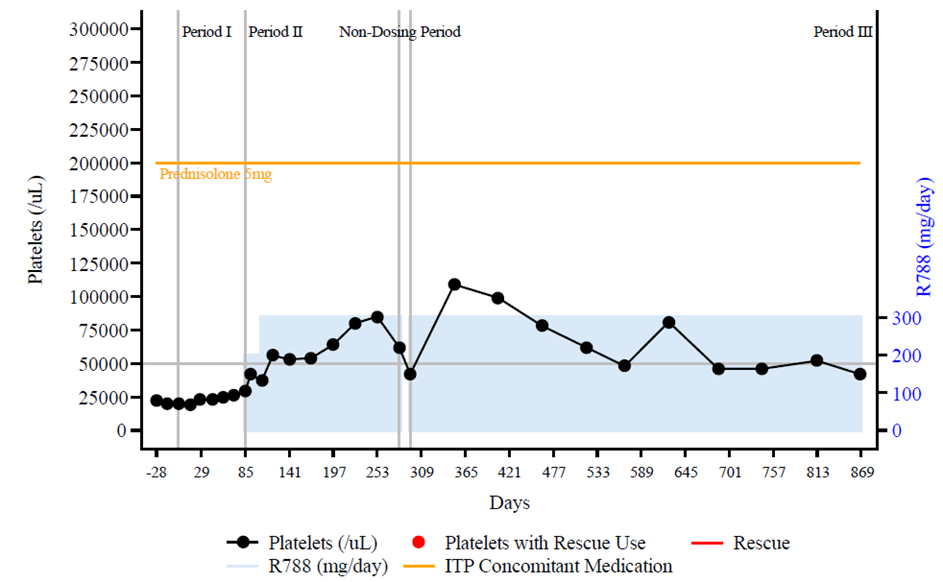


This patient used 5 mg prednisolone throughout the study.

Patient 17


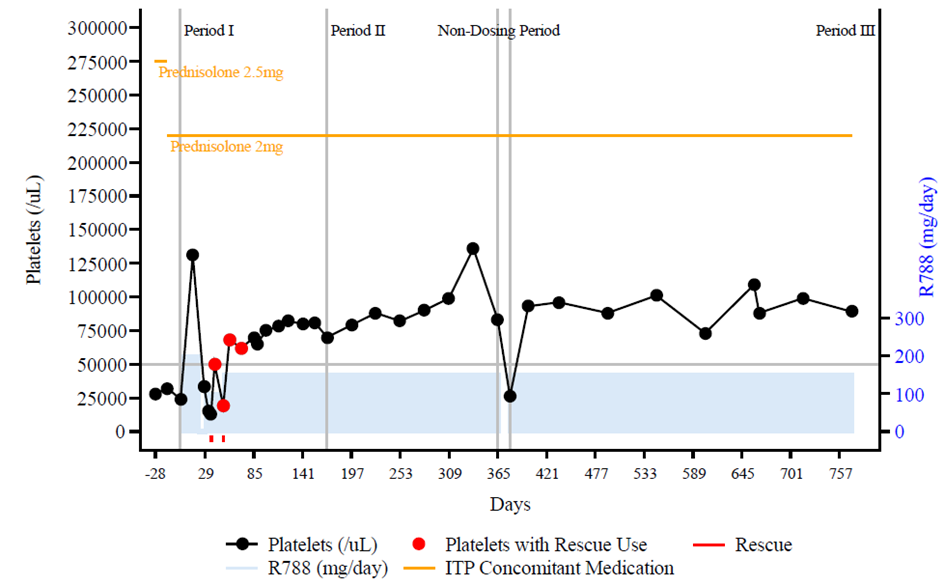


This patient used 2.5 mg prednisolone from day −28 to −15 and 2 mg from day −14 to 771.

Patient 18


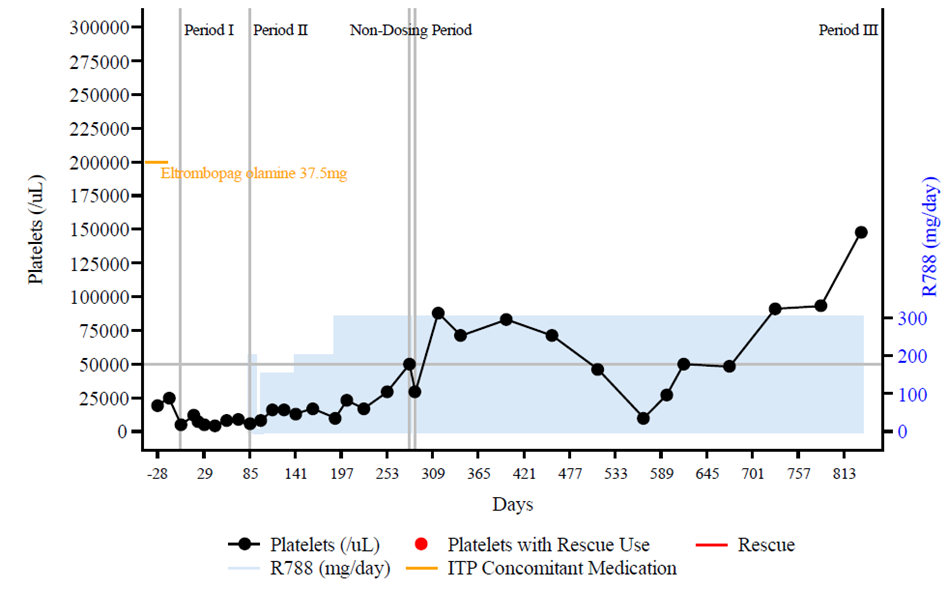


Patient 19


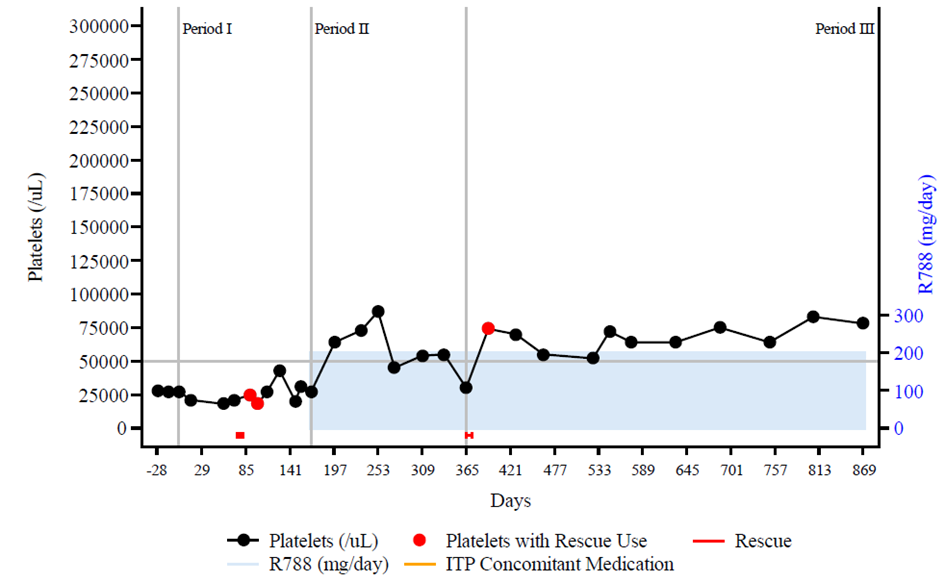


Patient 20


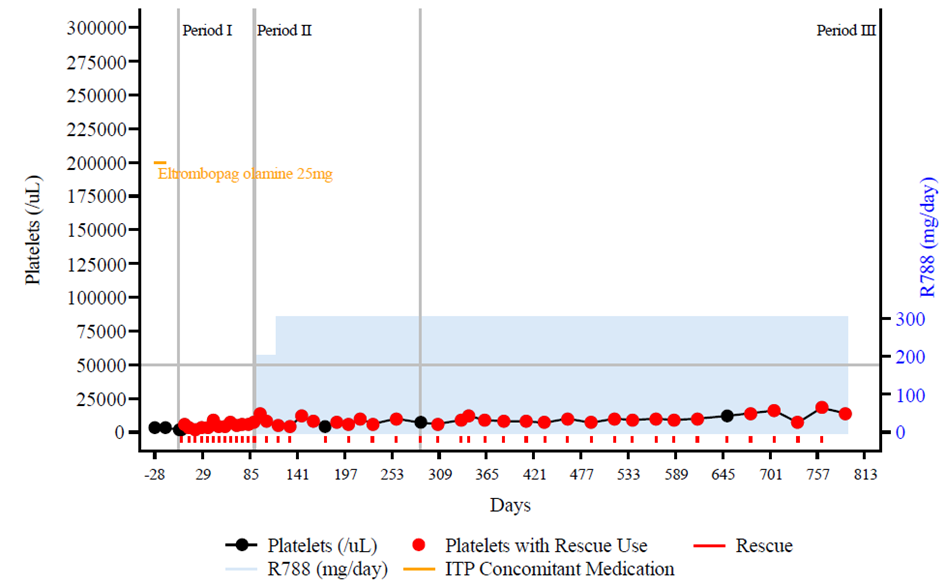


Patient 21


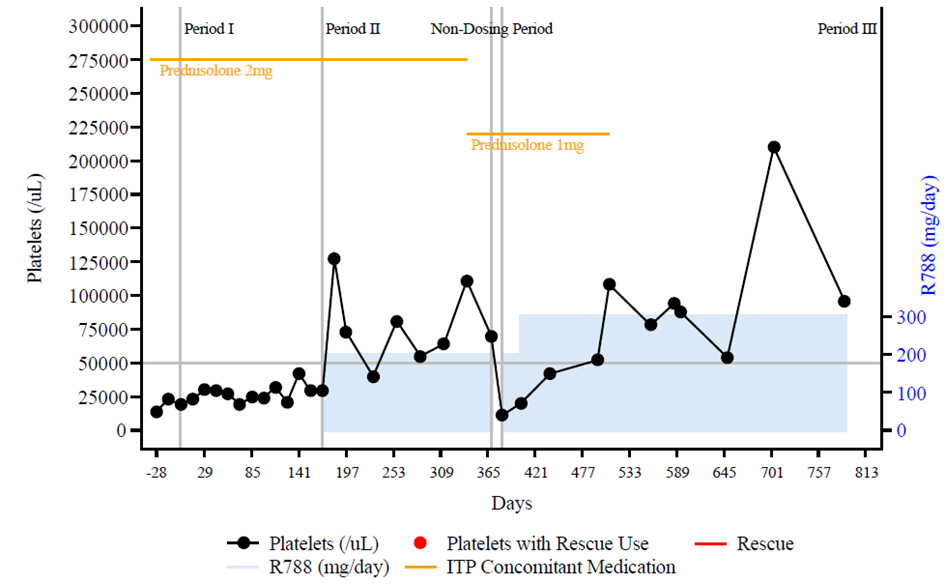


This patient used 2 mg prednisolone from day −35 to 340 and 1 mg from day 341 to 509. Thereafter, the patient discontinued prednisolone.

Patient 22


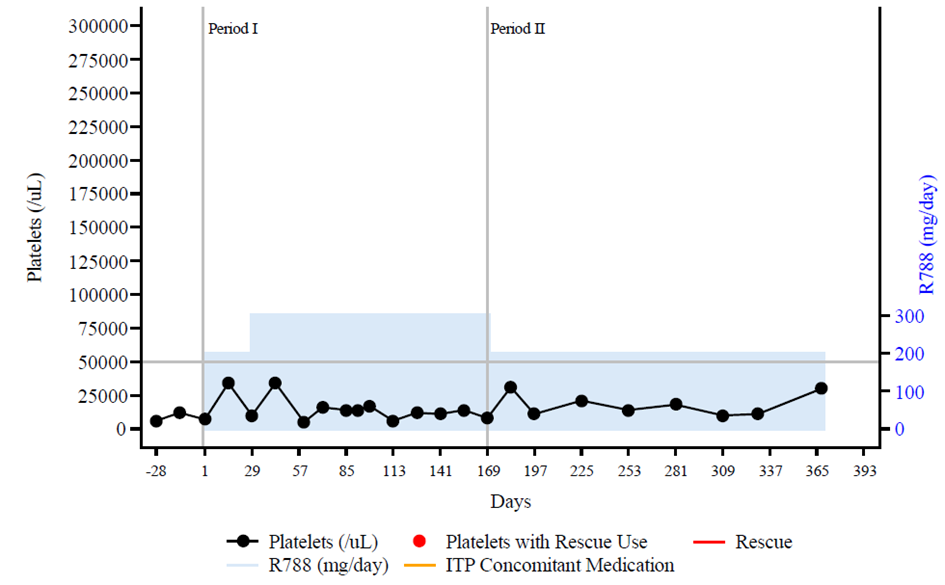


Patient 23


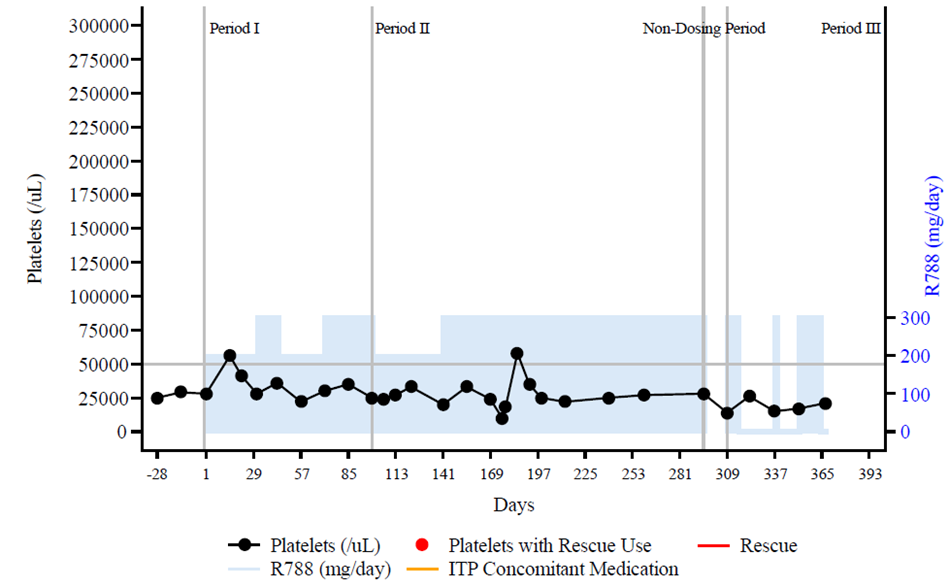


Patient 24


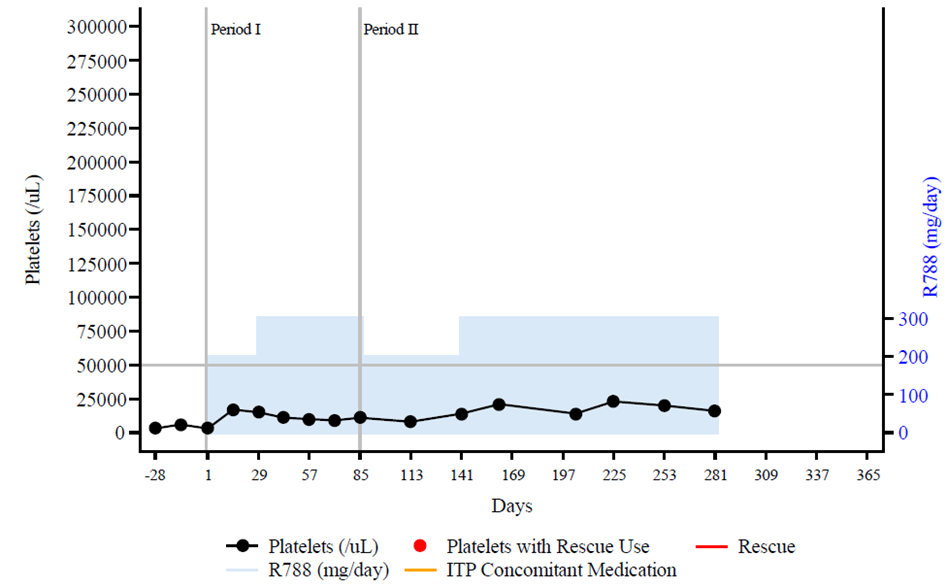


Patient 25


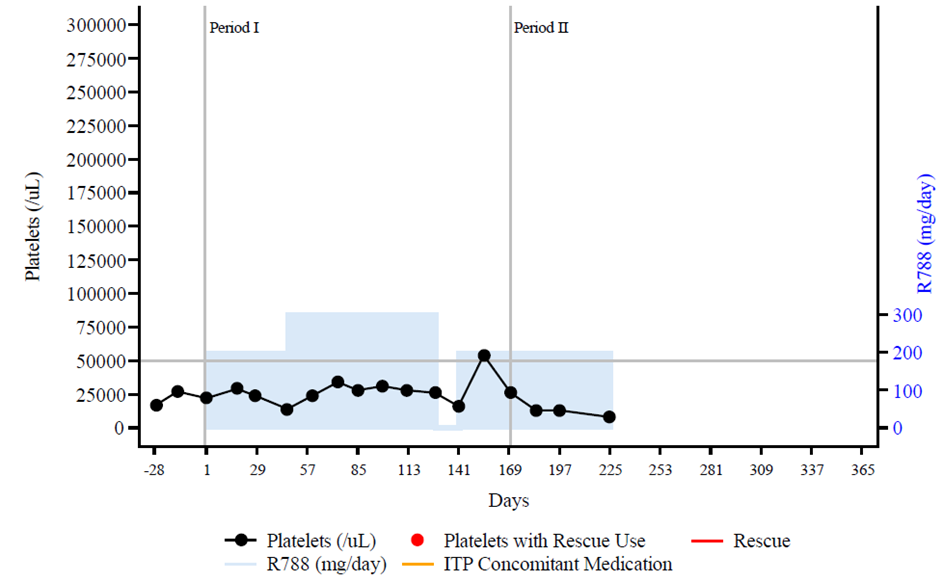


Patient 26


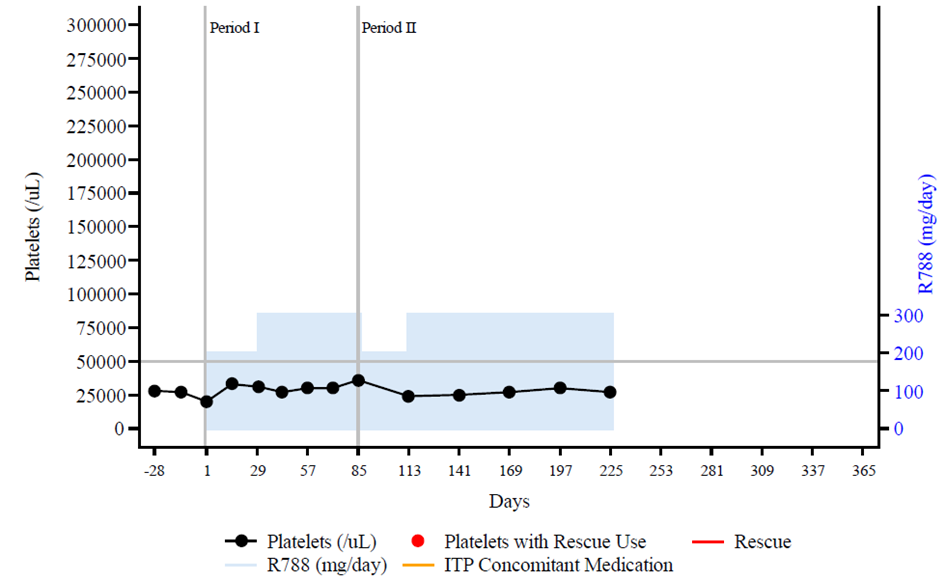


Patient 27


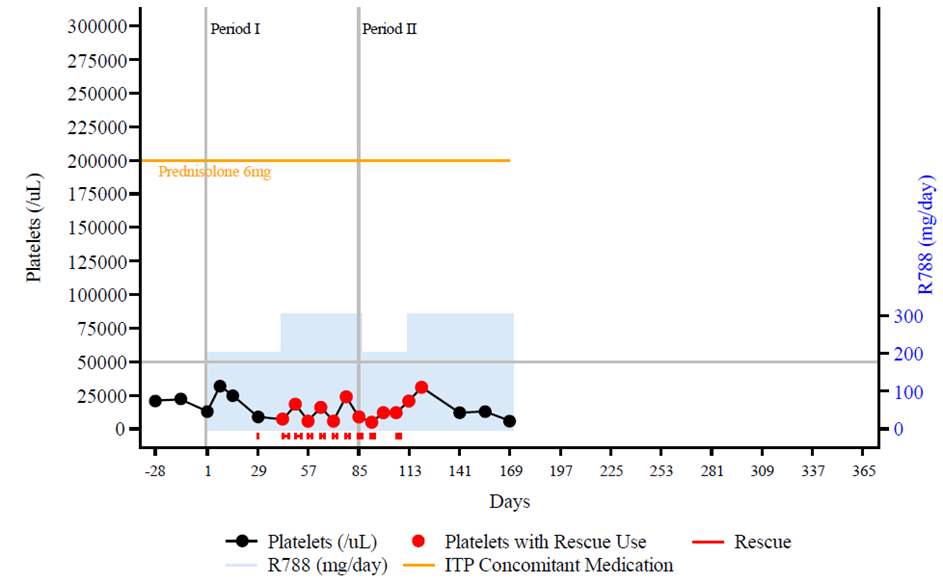


This patient used 6 mg prednisolone throughout the study.

Patient 28


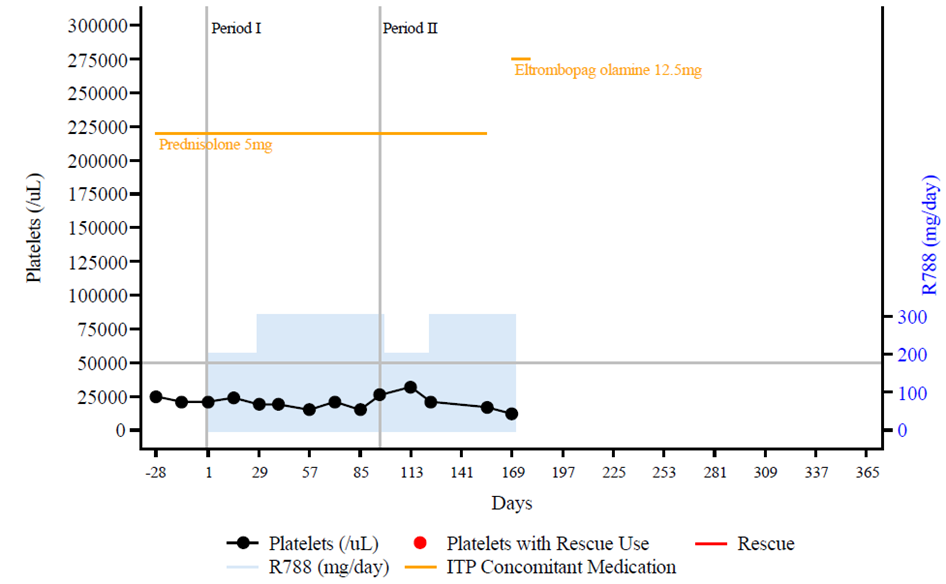


This patient used 5 mg prednisolone from day −28 to 155.

Patient 29


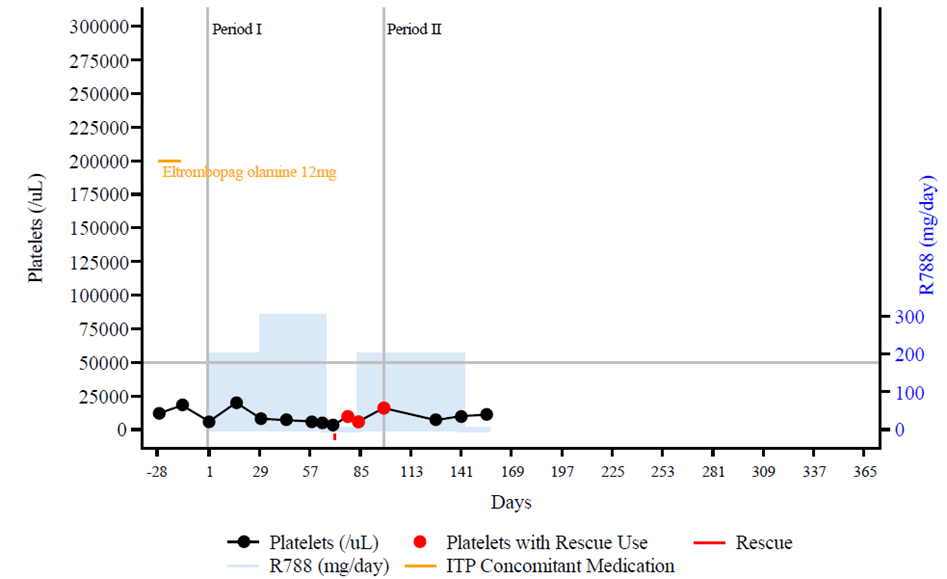


Patient 30


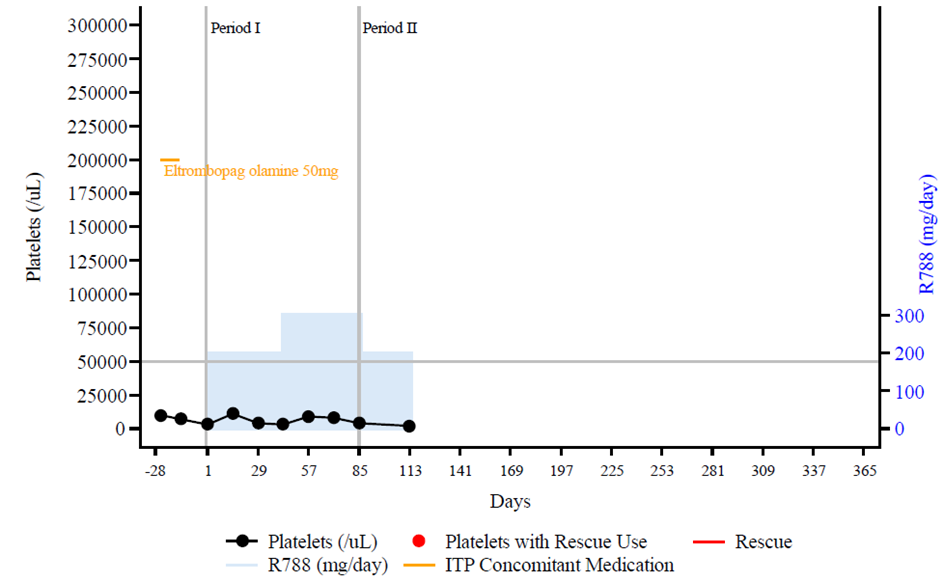


Patient 31


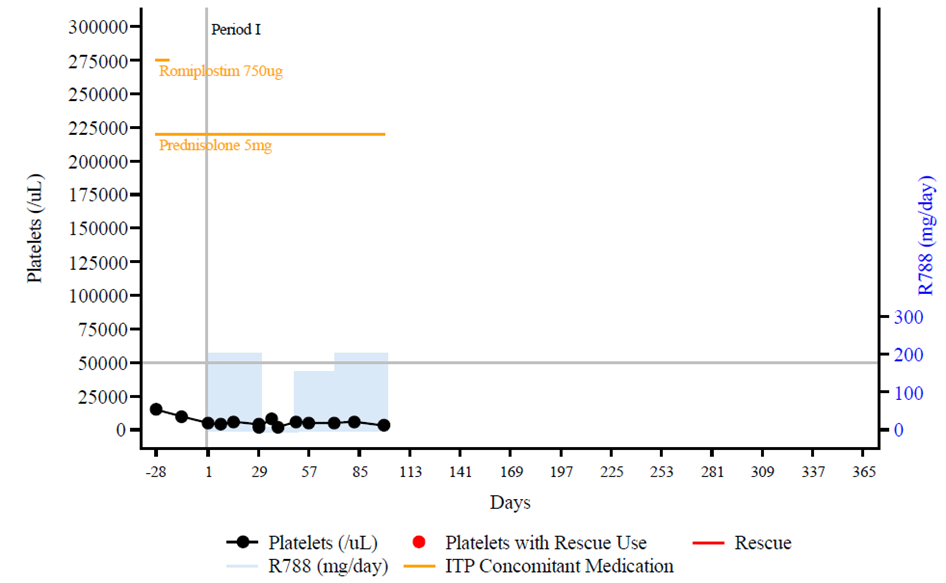


This patient used 5 mg prednisolone throughout the study.

Patient 32


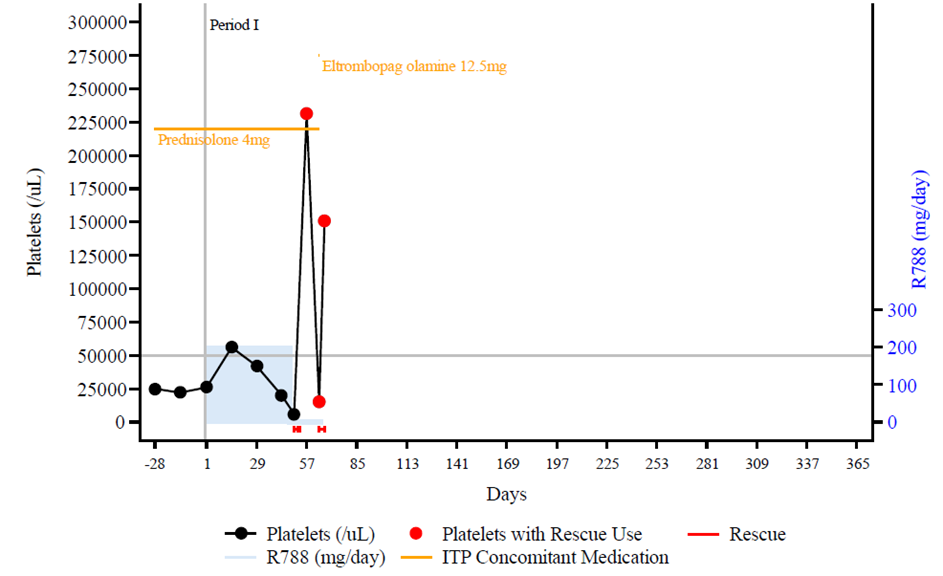


This patient used 4 mg prednisolone throughout the study.

Patient 33


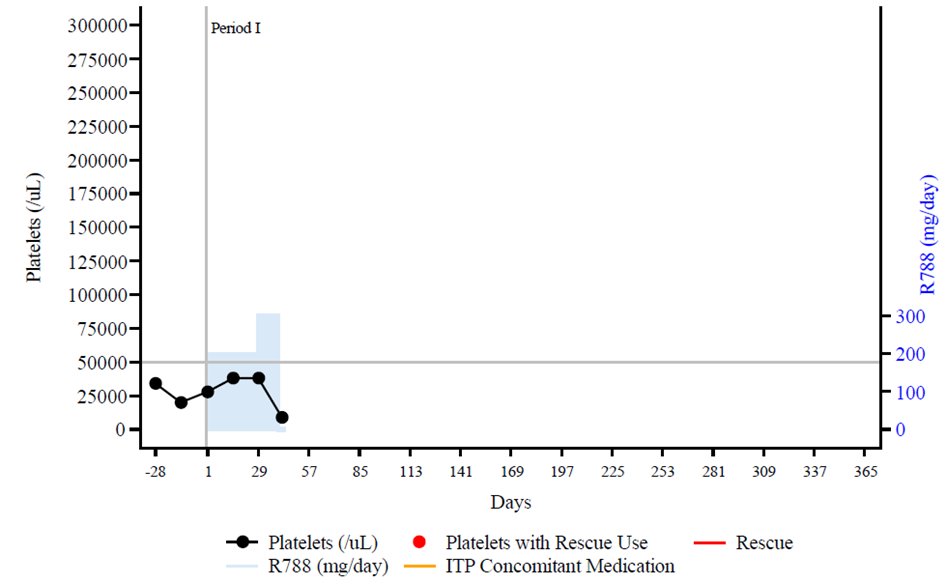

Supplement: Supplementary file 1 — Supplementary file1 (DOCX 3688 KB) [file 12185_2025_3924_MOESM1_ESM.docx]
